# An improved joint non-negative matrix factorization for identifying surgical treatment timing of neonatal necrotizing enterocolitis

**DOI:** 10.17305/bjbms.2022.7046

**Published:** 2022-05-15

**Authors:** Guoqiang Qi, Shoujiang Huang, Dengming Lai, Jing Li, Yonggen Zhao, Chen Shen, Jian Huang, Tianmei Liu, Kai Wei, Jinfa Tou, Qiang Shu, Gang Yu

**Affiliations:** 1 Department of Data and Information, The Children’s Hospital, Zhejiang University School of Medicine, Hangzhou, China; 2 Sino-Finland Joint AI Laboratory for Child Health of Zhejiang Province, Hangzhou, China; 3 National Clinical Research Center for Child Health, Hangzhou, China; 4 Department of Neonatal Surgery, The Children’s Hospital, Zhejiang University School of Medicine, Hangzhou, China; 5 Department of Radiology, The Children’s Hospital, Zhejiang University School of Medicine, Hangzhou, China; 6 Department of Electronic Engineering, College of Information Engineering, Shanghai Maritime University, Shanghai, China

**Keywords:** Neonatal necrotizing enterocolitis, joint non-negative matrix factorization, surgical indications, multimodal clinical data

## Abstract

Neonatal necrotizing enterocolitis is a severe neonatal intestinal disease. Timely, the identification of surgical indications is essential for newborns to seek the best time for treatment and improve prognosis. This paper attempts to establish an algorithm model based on multimodal clinical data to determine the features of surgical indications and construct an auxiliary diagnosis model. The proposed algorithm adds hypergraph constraints on the two modal data based on Joint Non-negative Matrix Factorization, aiming to mine the higher-order correlations of the two data features. In addition, the adjacency matrix of the two kinds of data is used as a network regularization constraint to prevent overfitting. Orthogonal and L1-norm regulations were introduced to avoid feature redundancy and perform feature selection, respectively, and confirmed 14 clinical features. Finally, we used three classifiers, random forest, support vector machine, and logistic regression, to perform binary classification of patients requiring surgery. The results show that when the features selected by the proposed algorithm model are classified by random forest, the area under the ROC curve is 0.8, which has high prediction accuracy.

## INTRODUCTION

Neonatal necrotizing enterocolitis (NEC) is one of the causes of neonatal death. Surviving newborns are likely to have intestinal stenosis, short bowel syndrome, and even short bowel syndrome. There are many serious sequelae, such as abnormal nervous system development [1]. About 2-5% of infants admitted to a neonatal intensive care unit (NICU) will have NEC, of which 20-40% require surgical intervention, and the mortality rate ranges from 20% to 35% [2,3]. The treatment for newborns with NEC is mainly divided into conventional medical treatment and surgical operation. Surgical treatment should be considered if conservative medical treatment fails or the disease progresses [4,5]. At present, intestinal perforation is the only absolute indication for the NEC surgery, and other indications are controversial [6-8].

Abdominal plain radiographs and clinical tests play an essential role in determining the timing of NEC surgical intervention. Abdominal plain radiographs have high specificity for NEC, but low sensitivity, and can only assist doctors in decision-making to a certain extent. It is necessary to make a timely diagnosis of NEC in combination with clinical test results. General clinical examination includes five types of biochemical analysis, blood routine, and blood gas analysis. Comprehensive analysis of whether the newborns need surgery through plain abdominal film and clinical study is significant for their prognosis and survival rate.

Joint non-negative matrix factorization (JNMF) [9] has been widely used in multimodal data fusion. It works by mapping the features of different modalities to a shared space where other heterogeneous variables are clustered in different directions. Based on this algorithm, many scholars have innovated to adapt to data types of different backgrounds and modalities. Deng *et al*. [10] applied the algorithm to the imaging genetics of lung adenocarcinoma by fusing the characteristics of case images, copy number variation, and DNA methylation data and adding network regularization constraints based on the JNMF algorithm. To improve the degree of association between different data, orthogonal constraints are added to remove redundant features and reduce the algorithm’s time complexity.

On this basis, this paper makes further innovations to adapt to NEC’s abdominal plain film data and clinical data. A hypergraph-based multi-constraint joint non-negative matrix factorization (HB-MCJNMF) is proposed, which innovatively uses hypergraphs to mine higher-order relationships between two data features. In addition, the algorithm was induced to mine features that are diagnostically meaningful for both diagnostic groups by introducing the patient’s diagnostic status (whether or not to undergo surgery). Experimental results show that the proposed HB-MCJNMF algorithm can identify specific association patterns of different diagnostic groups and mine clinical features with diagnostic significance. Area Under Curve (AUC) is defined as the area under the Receiver Operators Characteristic (ROC) curve enclosed by the coordinate axis. We performed classification using random forests, support vector machines, and logistic regression. Among them, the AUC for classification using random forest reaches 0.8.

## MATERIALS AND METHODS

### Data sources

This study was approved by the Medical Ethics Committee of the Children’s Hospital (Approval Letter of IRB/EC, 2022-IRB-030) and waived the need for written informed consent from patients, as long as patient data remained anonymous. All of the methods were carried out by the Declaration of Helsinki.

This paper retrospectively collected the clinical diagnosis and treatment data of 45 newborns with NEC from a children’s Hospital, including abdominal plain film and test data (biochemical Wuchang, blood routine and blood gas). Abdominal plain film was marked by radiologists with at least 8 years of experience. Standard abdominal X-ray plain film requirements are as follows: (1) The baby lay on his back on the stage and the nurse raised his arms and clamped his head and hands together; (2) keep the head, shoulders, and knees of the newborns close to the photography stage, and the middle of the body faces the center line of the table to ensure that the body position will not be skewed; (3) align the center line with the midpoint of the connecting line between xiphoid process and pubic symphysis, and vertically ingest the detector; and (4) the projection field shall be as small as possible, with the upper edge including the diaphragmatic surface and the lower edge including the pubic symphysis, so as to reduce the radiation dose of scattered rays.

Newborns were classified according to birth weight: (1) There were five extremely low birth weight newborns (birth weight <1000 g); (2) there were 11 very low birth weight newborns (1000 g ≤ birth weight < 1500 g); (3) there were 19 low birth weight newborns (1500 g ≤ birth weight < 2500 g); and (4) there were 10 normal birth weight newborns (2500 g ≤ birth weight < 4000 g). Newborns were classified according to gestational age: (1) There were eight term infant newborns (37 weeks ≤ gestational age < 42 weeks) and (2) there were 37 preterm infants (weeks gestational age < 37 weeks). Newborns were classified according to the age of weeks after birth: (1) There were 15 early newborns (age < 1 week). (2) There were 25 late newborns (1 week ≤ age < 4 weeks). (3) There were five other newborns (4 weeks ≤ age). Newborns were classified according to gender: There were 25 male and ten female newborns. There were 37 newborns delivered naturally and eight newborns delivered by cesarean section. There were 31 newborns who were twins and 14 newborns who were singletons.

### Data preprocessing

For the abdominal plain film image data, we used the pyradiomics package to extract the radionics features of the original image lesions including first order, first shape, and several texture features, including first-order statistics, first shape statistics, Gray-Level Co-Occurrence Matrix (GLCM), Gray-Level Run-Length Matrix (GLRLM), Gray-Level Size Zone Matrix (GLSZM), Gray-Level Dependence Matrix (GLDM), and Neighborhood Gray Tone Difference Matrix (NGTDM). Finally, 115 radiomic features were obtained.

For clinical data, we obtained a total of 79 indicators that are clinically closely related to NEC, and we used the *l_2_* norm to standardize the two kinds of data to ensure the non-negativity of the input data. Finally, we obtained two feature matrices *X_1_* ∈ *R*^45×115^ and *X_2_* ∈ *R*^45×79^, corresponding to plain abdominal film and clinical examination data, respectively.

### Method

**FIGURE 1:**
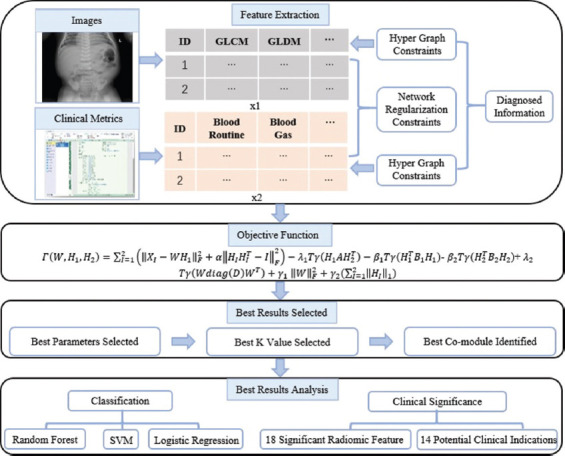
The overall flow chart of the construction of the diagnostic model in this paper.

This section describes the fusion of radionics and clinical features to identify features relevant to NEC diagnosis. As shown in Figure 1, we detail the overall pipeline of our algorithm. First, we extract features from two different modal data and organize them into two feature matrices. Each row represents a sample, and each column represents a sample feature. To further extract higher-order parts of the data, we introduce hypergraph constraints. In addition, to further strengthen the correlation of the two data, we add the adjacency matrix of the two data features as the network regularization constraint. Finally, to dig deeper into the diagnostically meaningful features of the two types of patients, we included the diagnostic information of the two types of patients. The performance of the proposed algorithm is verified using a variety of classifiers for classification through correlation analysis of elements in essential modules.

### JNMF

*X_i_*∈*R^n×pi^* denotes the original matrix of different modes, *W_i_*∈*R^n×k^* denotes the decomposed base matrix, and *H_I_*∈*R^k×pi^* denotes the decomposed original matrix Coefficient Matrices. To extract common modules among the three data matrices, the JNMF algorithm decomposes *X_i_* into a common basis matrix *W* and multiple coefficient matrices *H_i_* (*i*=1,2,…):


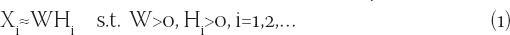


Define its objective function as follows:


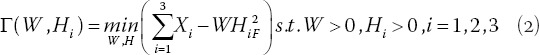


### Hypergraph learning

Unlike traditional graph methods, hypergraphs can connect more than two vertices through hyperedges, that is, an extension of the hypergraph simple graph. Therefore, we can use hypergraph to mine the higher-order relationships between the features of each modality data for modeling [11,12].

Considering that the relevant content of hypergraph representation has been given in [13], in this paper, we continue to explore the representation of symbols in hypergraph, as described below. We let *G* (*V, E, a*) represent the hypergraph, where *V* is the set of vertices, *E* is the set of hyperedges, and *a* is the set of weights of the hyperedges. Each hyperedge *e_i_* (*i*=1,…,*N_e_*) is assigned a weight *a* (*e_i_*). For a hypergraph *G*, we define its association matrix *H*. *H* is used to represent the relationship between hyperedges and vertices. For example, the *i*^th^ row and *j*^th^ column of *H* represent, whether the *j*^th^ hyperedge represents the *i*^th^ vertex. *H* is defined as the following representation:


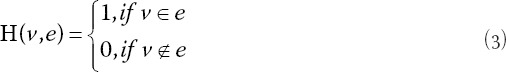


Further, we can get the degree matrix of each edge *v*.


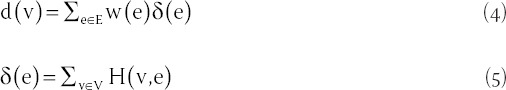


Furthermore, we use D*_v_* and D*_e_* to represent the diagonal matrices of vertices and hyperedges, respectively. To simplify the representation in the formulation, in the following, we define the hypergraph Laplace using simple Laplace [11].


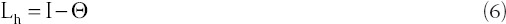


Among them, *L_h_* represents the hypergraph Laplacian matrix, 
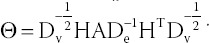
.

### HB-MCJNMF

In this section, to explore the association pattern between abdominal plain film image data and clinical examination data, and to mine significantly related expression modules, we introduce the hypergraph constraint mentioned in Section 2.2 into the JSNMNMF algorithm, aiming at the same model high-order associations between different features of state data.

*L_h1_* and *L_h2_* represent the hypergraph matrices of *X_1_* and *X_2_*, respectively. Next, we use *P*(*Hi*) (*i*=1,2,3) to rewrite the hypergraph constraints as follows:


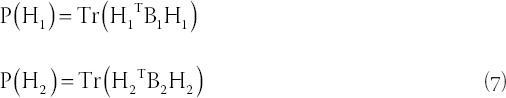


*B*_1_ and *B*_2_ represent the Laplace matrices of *X*_1_ and *X*_2_, respectively. So, we can determine that *B*_1_= *L_h1_* and *B*_2_= *L_h2_*. In addition, we add clinical diagnostic information *D* (whether or not to perform surgery) to induce the algorithm to pick out the differential features of the two categories. Specifically, it is coded as 1 for the non-surgical label and 2 for the surgical label. Furthermore, we get the objective function of the HB-MCJNMF algorithm:


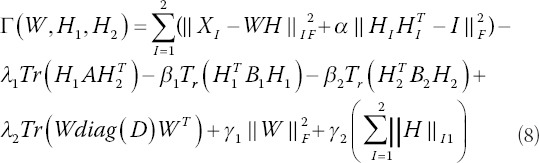


Among them, *A* represents the adjacency matrix of the two kinds of data, and *diag*(*D*) represents the diagonal matrix of *D*. Suppose *φ_ij_* and ϕ*_ij_^I^* are Lagrangian multipliers of *W_ij_* ≥ 0 and (*H_I_*)*_ij_* ≥ 0, respectively, then the Lagrangian function is expressed as:


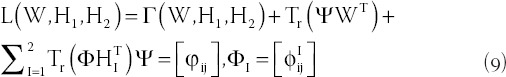


The partial derivatives of *L* with respect to *W* and *H_I_* are obtained, respectively:


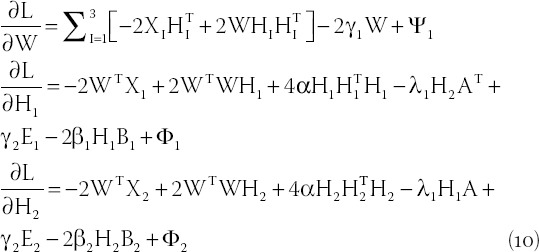


Among them, *E_i_* (*i*=1,2,3) is a matrix whose elements are all 1. According to the Karush-Kuhn-Tucker) condition, *φ_ij_* *W_ij_* = 0 and *Φ_ij_^I^* (*H_I_*)*_ij_* = 0, we can get the equations for *W_ij_* and (H_I_)_ij_:


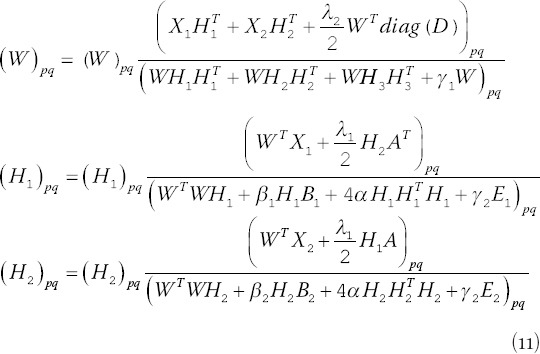


### Module member selection

Through the iterative update of the above algorithm, the feature matrix *X*_1_ of the abdominal plain film and the feature matrix *X*_2_ of the clinical data are transformed into the basis matrix *W* and the coefficient matrices *H*_1_ and *H*_2_. To find the weights corresponding to the salient features of each row of *W*, we use z-score to extract the coefficients of each row of *H_I_*. It is defined as follows:


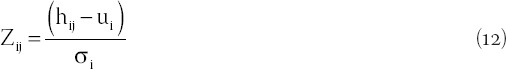


where *h_ij_* represents the element in *H_I_*, *u_i_* represents the mean of feature in *H_I_*, and σ*_i_* represents the standard deviation. Next, to determine module membership, we set a threshold *T*, and if the element’s z-score is greater than the set threshold *T*, it is considered eligible for assignment to the module.

### Parameter selection

It can be seen from equation (11) that for the HB-MCJNMF algorithm, *W*, *H*_1_, and *H*_2_ need to be initialized. The previous research [10] has confirmed that the initialization using the singular value decomposition strategy can make the algorithm perform better. Therefore, SVD initialization is still used in this paper.

**FIGURE 2:**
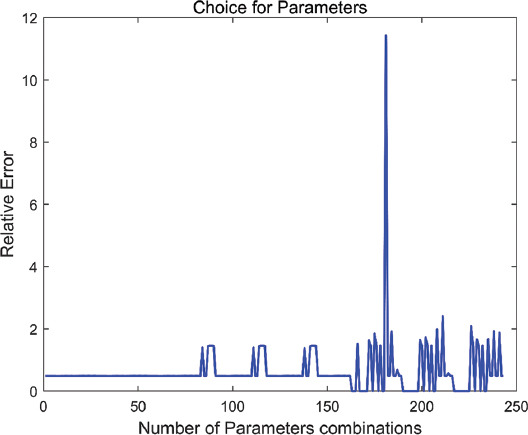
Reconstruction errors obtained under different parameter combinations.

The different choice of parameters will affect the objective function value of the JNMF algorithm. Therefore, we first fix the K value to 15 and adjust *α*, *λ_i_*, *β_i_*, and *γ_i_* (*i*=1,2) according to the finite set of (0.001, 0.01, 0.1). Considering the time cost, we make *λ*_1_=*λ*_2_, *β*_1_=*β*_2_. We show the reconstruction error obtained by sequentially substituting 243 sets of regularization parameters into the algorithm in the Figure 2.

As shown in the Figure 2, since the objective function value does not converge under certain parameter combinations, we set the reconstruction error in this case to 0 in Figure 2. The reconstruction error was the smallest at 0.2936 under the 61^st^ parameter combination. We use the following parameter combinations: α=0.1, *λ_i_*=0.001, *β_i_*=0.1, *γ*_1_=0.001, *γ*_2_=0.1.

**FIGURE 3:**
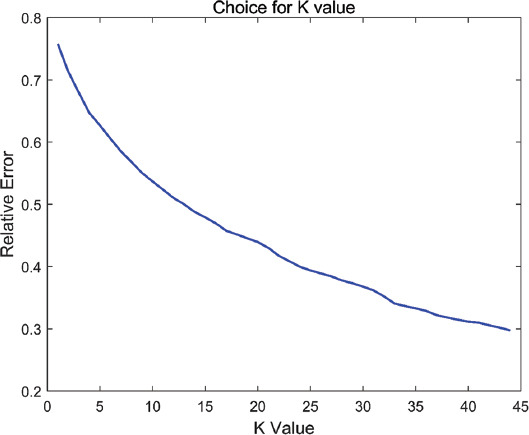
Reconstruction errors obtained under different K values.

After selecting the best parameter combination, we selected the number of coexpression modules *K*. Since the number of samples in this paper is 45, the upper limit of *K* is set to 45, and the lower limit is 2, as shown in the Figure 3.

We found that as the value of K increased, the reconstruction error decreased, so we took 45 coexpression modules. According to the selection of the above parameters and K value, we obtained the decomposition results *W*, *H*_1_, *H*_2_, and 45 coexpression modules of the original matrices *X*_1_ and *X*_2_. The Pearson correlation coefficient between *X*_1_ and *WH*_1_ was 0.9848, and the Pearson correlation coefficient between *X*_2_ and *WH*_2_ was 0.9584.

**FIGURE 4:**
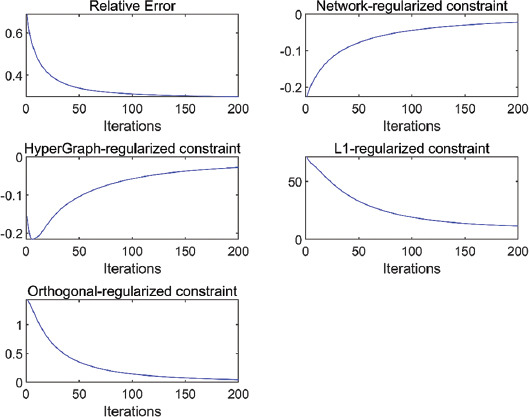
The relative error of the algorithm and the change trend of the function value of each constraint with the increase of the number of iterations under the condition of optimal parameters.

Next, we show the function values of the different constraints in the proposed algorithm as a function of the number of iterations, as shown in the Figure 4. It can be seen from Figure 4 that both the relative error and the constraints added based on the JNMF algorithm tend to converge with the increase of the number of iterations, which explain the stability of the proposed algorithm to a certain extent.

**FIGURE 5:**
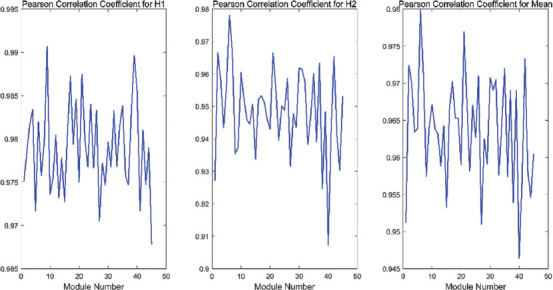
The three subgraphs from the left to right represent the Pearson correlation coefficients of the original matrices X_1m_ and W_1m_ H_1m_ in different modules, the Pearson correlation coefficients of the original matrices X_2m_ and W_2m_ H_2m_, and the mean of the coefficients.

For the 45 coexpression modules, we calculated the Pearson correlation coefficients of the original matrices (*X*_1*m*_ and *X*_2*m*_) and the reconstructed matrices (*W*_1*m*_ *H*_1m_ and *W*_2*m*_ *H*_2*m*_) in each module, respectively, *m*=1,2,…,4,5 represents the module number. In addition, we also calculated the mean of the two Pearson correlation coefficients for each module, as shown in the Figure 5.

## RESULTS

**FIGURE 6:**
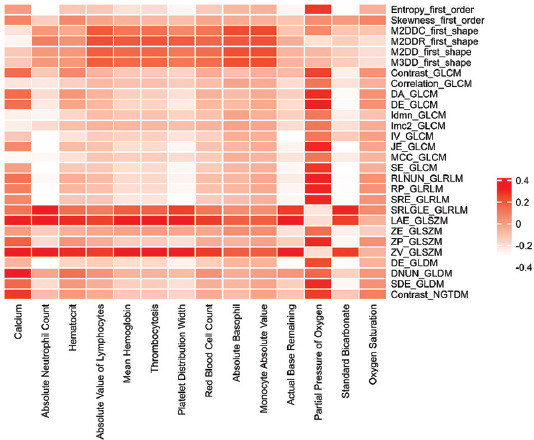
Heat map of the correlation between radiomics’ features and clinical features.

As shown in Figure 6, Module 6 has the highest average Pearson correlation coefficient, so we choose Module 6 for subsequent analysis. A total of 18 significant radiomic feature and 14 potential clinical indications were obtained. We present the salient features and descriptions of the two types of data selected by Module 6 in Tables 1 and 2 below, respectively. For the description of the radiomic features selected by the HB-MCJNMF algorithm in Table 1, please refer to https://pyradiomics.readthedocs.io/en/latest/.

**TABLE 1:**
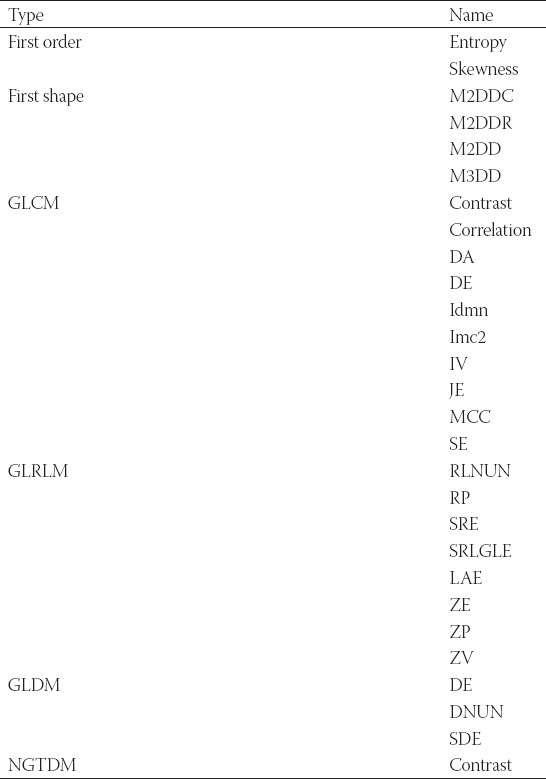
Radiomics’ salient features and their descriptions in Module 6

**TABLE 2:**
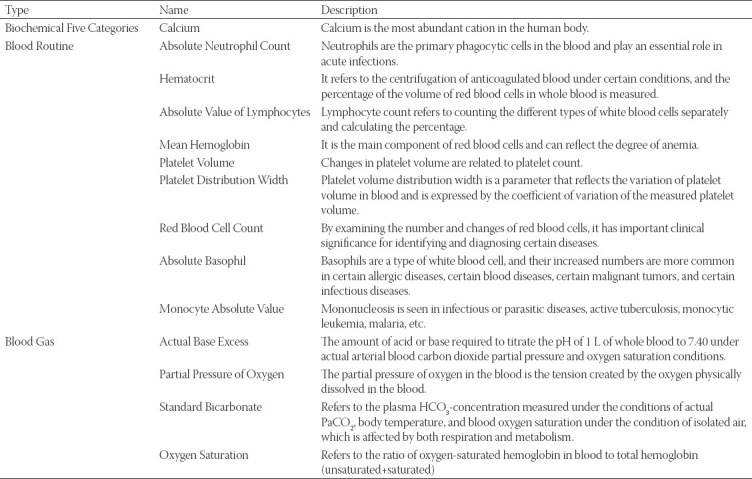
Clinically significant features and their descriptions in Module 6

In addition, we plotted a heat map of selected radiomics and clinical features to demonstrate the correlation between the two modalities of data, as shown in the Figure 6.

It shown in Figure 6 that most of the features have strong correlations, which indicates that the features selected by the algorithm are highly representative and can be effectively used as diagnostic features. At present, there is no correlation study on the data of these two modalities of NEC. The significant association features, we found, could not be further confirmed. To facilitate future reference for researchers, we list the top ten feature pairs with significant correlation in Figure 6 (top 10 feature pairs after the absolute value of the Pearson correlation coefficient), as shown in Table 3. It is shown in Table 3 that ZV_GLSZM, LAE_GLSZM may be associated with multiple clinical features.

**TABLE 3:**
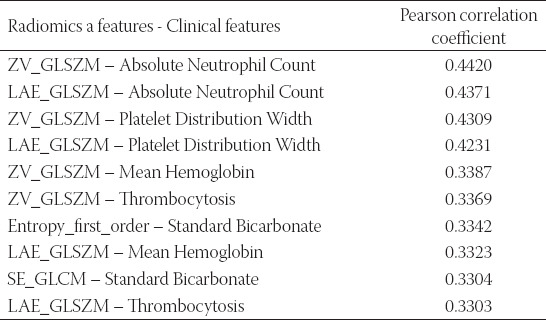
Clinically significant features and their descriptions in Module 6

**FIGURE 7:**
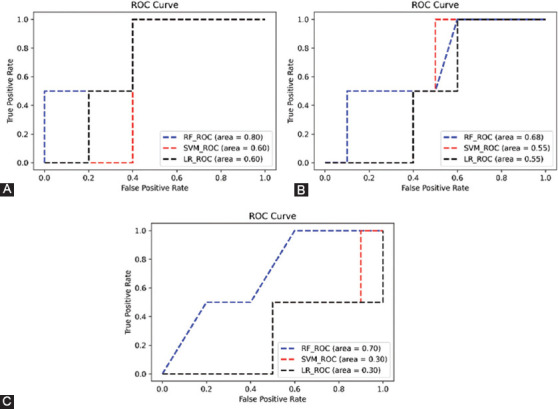
(A) ROC curves for classification using algorithmically selected fused features; (B) ROC curves for classification using radiomic features alone; and (C) ROC curves for classification using clinical features alone.

To further explore whether the features selected by the algorithm were diagnostic, we used random forest, SVM, and logistic regression to classify patients for surgery or not, respectively. We use grid search to find the optimal classification parameters for each classifier before performing the classification task. During classification, the non-randomness of the experiment is guaranteed by setting a random seed, in which 75% of the total number of samples are used as the training set. About 25% of the data are used as the test set. Figure 7A-C show the test set ROC curves of the fusion feature selected by the algorithm, imaging data alone, and clinical data alone.

As shown in Figure 7, the area under the ROC curve of the fusion features selected by the algorithm using the random forest classifier for classification reaches 0.8, which is much higher than the classification accuracy of the other two schemes. This confirms the feature selection ability of the algorithm and the robustness of the algorithm.

## DISCUSSION

In this section, we analyze the selected features from the perspective of clinical significance. From the results of radiomics feature selection, most of the features selected by the algorithm come from three texture features, including GLCM, GLRLM, and GLDM. The GLCM is used to describe the gray level relationship between a pixel and other pixels in an image. In medical imaging, this feature has been widely used in disease classification and diagnosis and has achieved good classification results [14]. Similar to GLCM, GLRLM [15], and GLDM [16] also play an important role in image classification.

In this section, we analyze the selected features from clinical significance. From the results of radionics feature selection, most of the elements chosen by the algorithm come from three texture features, including GLCM, GLRLM, and GLDM. The gray level cooccurrence matrix (GLCM) describes the gray level relationship between a pixel and other pixels in an image. This feature has been widely used in disease classification and diagnosis in medical imaging and has achieved good classification results [14]. Like GLCM, GLRLM [15], and GLDM [16] also play an important role in image classification.

Activation of calcium/calmodulin-dependent protein kinase IV (CaMKIV) has been shown to increase Dextran Sulfate Sodium-induced intestinal injury and inhibit epithelial cell proliferation in mice with colitis. One experiment [17] demonstrated that the expression and activation of CaMKIV were upregulated in NEC mice, suggesting a potential contributing factor in the pathogenesis of NEC. Endotoxemia (ETX) is the most severe in newborns with NEC. Studies have found that ETX is negatively correlated with absolute neutrophil count [18], which suggests that absolute neutrophil count is a potential clinical indicator of NEC. The literature [19] confirmed the correlation between NEC mortality in newborns and Hematocrit and Absolute Value of Lymphocytes.

In a retrospective cohort study, among infants most prone to NEC, temporarily maintaining higher baseline hemoglobin may have a protective effect [20], suggesting that the mean of Hemoglobin may have some value in the early diagnosis of NEC. To develop a preclinical model of NEC-associated thrombocytopenia, the researchers measured serial platelet counts in mouse pups with trinitrobenzene sulfonic acid-induced NEC-like lesions results showed immature platelet counts containing the breadth of platelet distribution. Platelet fraction is associated with NEC-like injury in mice [21]. One study used Cox regression models to investigate factors associated with NEC and mortality, and they found a significant relationship between blood counts and increased mortality in NEC [22], recording clinical features and absolute monocyte counts, using ROC tested, the diagnostic accuracy of AMC values [23] suggests that lower (85-89%) oxygen saturation target levels increase NEC risk compared with higher (91-95%) oxygen saturation target levels.

To further confirm the clinical significance of the selected clinical features, we compared the 14 clinical features with symptoms in seven clinical metrics of metabolic derangement (MD7) associated with the three types of tests (biochemical five, blood routine, and blood gas) selected in this paper. We found that the four metrics related to these three examinations in MD7 were acidosis, hyponatremia, thrombocytopenia, and neutropenia. By consulting the diagnostic basis of these four metrics, we found that most of the selected 14 characteristics were closely related to the diagnosis of the four metrics.

The data of the two different modalities used in this paper will introduce some noise during the processing. HB-MCJNMF algorithm proposed in this paper can effectively improve the anti-noise performance of the algorithm. To test this idea, we use the original JNMF algorithm and the HB-MCJNMF algorithm to compare the algorithm performance on the real patient dataset and the synthetic dataset, respectively.

In real data, we set the number of iterations for both algorithms to be 100 and then compare the reconstruction errors and the common *l_1_* norm of the two algorithms. As shown in Table 4, the HB-MCJNMF algorithm obtains a smaller reconstruction error and *l_1_* norm, which, to some extent, confirms the performance advantage of the algorithm.

**TABLE 4:**
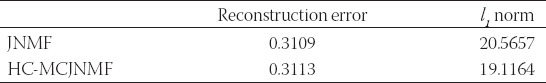
Comparison of algorithm performance between JNMF and HC-MCJNMF in real data

In synthetic data, we compare the anti-noise performance of the two algorithms. Specifically, we generate a dataset and set the total number of samples to 100, and the number of features for both modal data is 100. Furthermore, we set K=45. The following equations generate the basis matrix and the coefficient matrix *H_I_* (*I*=1,2,3).


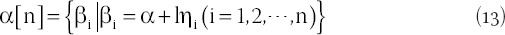


a is a matrix of random integers (1-10) obeying the uniform distribution *U*(1,10). *η_i_* and *l* represent Gaussian noise and its level, respectively. We present the reconstruction error and *l*_1_ norm of the two algorithms in Table 5. As shown in Table 5, the reconstruction errors of the proposed HB-MCJNMF algorithms are lower than those of JNMF as the noise increases. This confirms that the HB-MCJNMF algorithm has better anti-noise performance than JNMF and can effectively correlate radiomics and clinical data with certain noise.

**TABLE 5:**
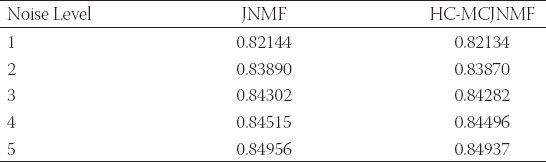
Comparison of algorithm performance between JNMF and HC-MCJNMF in synthetic data

## CONCLUSION

Based on the complementarity between data features of different modalities, this paper extracts radionics features and clinical features from plain abdominal films and clinical examinations of NEC patients for feature fusion and then constructs a diagnostic model. Among them, we propose an HB-JNMF algorithm for robust feature selection during feature fusion. We use three commonly used classifiers for diagnostic model construction in the classification part. Among them, the area under the ROC curve for classification using random forest reaches 0.8. The diagnostic model can assist physicians in making decisions about the need for surgery in patients with NEC. In addition, most of the clinical features found by the algorithm have also been confirmed by the previous studies. Physicians can refer to these significant clinical indicators when conducting NEC-related clinical examinations.

In the future, we will try to fuse features from more modalities, such as genomics data of NEC patients, to fuse more types of features to build a more comprehensive diagnostic model for NEC. In addition, collecting as many samples as possible is also beneficial for comprehensive feature fusion.
